# The Wnt Signaling Pathway in Diabetic Nephropathy

**DOI:** 10.3389/fcell.2021.701547

**Published:** 2022-01-04

**Authors:** Haiying Wang, Ran Zhang, Xinjie Wu, Yafen Chen, Wei Ji, Jingsuo Wang, Yawen Zhang, Yong Xia, Yiqun Tang, Jinxiang Yuan

**Affiliations:** ^1^ Department of Physiology, Jining Medical University, Jining, China; ^2^ Basic Medical School, Jining Medical University, Jining, China; ^3^ Key Laboratory of Precision Oncology of Shandong Higher Education, Institute of Precision Medicine, Jining Medical University, Jining, China; ^4^ Department of Clinical Pharmacy, School of Basic Medicine and Clinical Pharmacy, China Pharmaceutical University, Nanjing, China; ^5^ Collaborative Innovation Center, Jining Medical University, Jining, China

**Keywords:** diabetic nephropathy, Wnt, mesangial cell injury, podocyte injury, renal fibrosis

## Abstract

Diabetic nephropathy (DN) is a serious kidney-related complication of both type 1 and type 2 diabetes mellitus (T1DM, T2DM) and the second major cause of end-stage kidney disease. DN can lead to hypertension, edema, and proteinuria. In some cases, DN can even progress to kidney failure, a life-threatening condition. The precise etiology and pathogenesis of DN remain unknown, although multiple factors are believed to be involved. The main pathological manifestations of DN include mesangial expansion, thickening of the glomerular basement membrane, and podocyte injury. Eventually, these pathological manifestations will lead to glomerulosclerosis, thus affecting renal function. There is an urgent need to develop new strategies for the prevention and treatment of DN. Existing evidence shows that the Wnt signaling cascade plays a key role in regulating the development of DN. Previous studies focused on the role of the Wnt canonical signaling pathway in DN. Subsequently, accumulated evidence on the mechanism of the Wnt non-canonical signaling indicated that Wnt/Ca^2+^ and Wnt/PCP also have essential roles in the progression of DN. In this review, we summarize the specific mechanisms of Wnt signaling in the occurrence and development of DN in podocyte injury, mesangial cell injury, and renal fibrosis. Also, to elucidate the significance of the Wnt canonical pathway in the process of DN, we uncovered evidence supporting that both Wnt/PCP and Wnt/Ca^2+^ signaling are critical for DN development.

## Introduction

Globally, diabetic nephropathy (DN) is the most common form of secondary kidney disease that leads to end-stage renal disease (Kawanami et al., 2016). The main pathological and clinical features of DN are thickening of the glomerular basement membrane, the accumulation of mesangial cells, renal fibrosis, and proteinuria. However, the specific pathogenesis of DN has yet to be elucidated. The Wnt signaling pathway is an evolutionarily conserved cell-to-cell communication system that plays important roles in the inflammatory response, cell proliferation and differentiation during embryogenesis, and tissue homeostasis in adults. Pharmacological mechanisms, or genetic mutations, that lead to either low or high levels of activation in the Wnt signaling cascade have also been associated with a range of human diseases, including DN, renal fibrosis, kidney cancer, and acute renal failure. The treatment of DN based upon the findings of mechanistic studies has proven to be very challenging. Gaining a more comprehensive understanding of the precise relationship between the Wnt signaling pathways and DN will allow us to develop novel strategies for diagnostic procedures and personalized therapeutic interventions.

## The Wnt Signaling Pathway

Wnt is a class of secreted and cysteine-rich glycoproteins that was first identified in 1982 by Nusse et al. (Nusse_ and Varmus, 1982). The term “Wnt” is derived from the combination of two genes “*wingless*” and “*int-1*” that encode homologous proteins (Rijsewijk et al., 1987). The Wnt signaling pathway shows high levels of conservation across invertebrates and vertebrates (Kassumeh et al., 2021). The Wnt signaling pathway plays a significant role in embryonic development and oncogenesis and has been demonstrated to act as a crucial regulator in various diseases, including breast and prostate cancers, glioblastoma, and type 2 diabetes (Logan and Nusse, 2004; Komiya and Habas, 2008). The Wnt signaling pathway can be classified into two main pathways: a canonical β-catenin dependent pathway and a non-canonical β-catenin-independent pathway (Supplementary Figure S1). Wnt1, Wnt2, Wnt3, Wnt3a, Wnt8a, Wnt8b, Wnt10a and Wnt10b are common activators of the canonical pathway, whereas Wnt4, Wnt5a, Wnt5b, Wnt6, Wnt7a, Wnt7b and Wnt11 are common activators of non-canonical Wnt-signaling (Gajos Michniewicz and Czyz, 2020; O’Connell et al., 2009; Ackers et al., 2018). It is key to note that Wnt3a, Wnt5a and Wnt9b has been evidenced to be activated in both canonical and non-canonical Wnt pathways (Tu et al., 2007; Kestler and Kühl, 2008; He et al., 2015; Fu et al., 2016; Malik et al., 2020).

These two cascades can be activated by the binding of Wnt ligands to their Frizzled (Fzd) receptor (Routledge and Scholpp, 2019). In the absence of an extracellular Wnt stimulus, cytoplasmic β-catenin becomes trapped by a multi-protein “destruction complex” that includes adenomatous polyposis coli (APC), the scaffolding protein Axin, casein kinase 1 (CK1), and glycogen synthase kinase 3β (GSK-3β). After combining with the destruction complex, the β-catenin is sequentially phosphorylated by CK1 and then GSK-3β. The phosphorylated β-catenin is recognized by the E3 ubiquitin ligase β-transducing repeat-containing protein (β-TrCP), which then targets β-catenin for proteasomal degradation. In the presence of Wnt, the Wnt/β-catenin pathway is activated by the binding of Wnt ligands to the Fzd receptor and low-density lipoprotein receptor 5/6 (LRP5/6) co-receptor, resulting in the formation of a heterotrimer. The formation of the Wnt-Fzd-LRP complex, together with the recruitment of the scaffolding protein disheveled (Dvl), subsequently results in the phosphorylation of LRP6 and the activation and recruitment of the Axin complex to the receptors. These events lead to the inhibition of Axin-mediated β-catenin phosphorylation and degradation. Stabilized β-catenin then translocates from the cytoplasm to the nucleus where it binds to T cell factor and Lymphoid Enhancing Factor (TCF/LEF) and other transcription factors, thus leading to the activation of Wnt target genes (He et al., 2004; MacDonald et al., 2009; Clevers and Nusse, 2012; Stamos and Weis, 2013). Therefore, the canonical Wnt/β-catenin pathway plays an important role in regulating gene transcription (MacDonald et al., 2009).

The non-canonical Wnt signaling pathway can be further divided into two distinct branches: the planar cell polarity (PCP) pathway and the Wnt/Ca^2+^ pathway (Supplementary Figure S1). In the Wnt/PCP signaling pathway, the Wnt protein interacts with Fzd on the cell surface; then, Dvl activates Rac to activate downstream mitogen-activated protein 3 kinases (MAP3Ks) and mitogen-activated protein 2 kinases (MAP2Ks) to activate Jun N-terminal kinases (JNK). Dvl is connected to downstream Ras homologue gene-family member A (RhoA) and Rho-associated kinase (ROCK) *via* Dvl associated activator of morphogenesis 1 (Daam1); RhoA is also known to activate JNK. These events help regulate the cytoskeleton and gene expression (Pataki et al., 2015). In the Wnt/Ca^2+^ signaling pathway, the binding of the ligand to the Fzd leads to the activation of homotrimeric G protein (De, 2011) and activates phospholipase C (PLC). PLC leads to an increase in intracellular Ca^2+^, a reduction in guanosine 3′, 5′-cyclic phosphate (cGMP) levels, and the activation of calmodulin-dependent protein kinase II (CaMKII) or calcineurin (CalN) and PKC. CaMKII is an upstream protein kinase that phosphorylates cAMP response element binding protein (CREB). Transcription is initiated once CREB has been activated and CREB binding protein (CBP) has aggregated. CalN is a serine/threonine protein phosphatase that is dependent on Ca^2+^ and calmodulin. CalN stimulates an increase in intracellular calcium concentration that leads to the saturation of calmodulin and promotes the activation of calcineurin. The nuclear factor of activated T cells (NFAT) transcription factor is then activated in the cytoplasm and then dephosphorylated. Dephosphorylated NFAT translocates to the nucleus and then cooperates with other transcription factors to participate in calcium-induced gene expression (Molkentin, 2004; Kohn and Moon, 2005; Li et al., 2005). An overview of the Wnt signaling cascades is summarized in Supplementary Figure S1.

## The Wnt Signaling Pathway and Diabetes

Diabetes mellitus (DM) refers to a group of metabolic diseases that are characterized by hyperglycemia resulting from defects in insulin secretion, insulin action, or both (Testa et al., 2017). Most cases of diabetes fall into two broad categories in terms of etiopathogenesis (Fioretto et al., 2007). In one category, T1DM is caused by an absolute deficiency in insulin secretion due to the destruction of pancreatic islet β-cells. T1DM has a high incidence in children and adolescents (Li et al., 2017). The other category, T2DM is more prevalent and common in adults. T2DM is caused by a combination of resistance to insulin action (IR) and an inadequate compensatory insulin secretory response (Henning., 2018; Karatas et al., 2021). Diabetes is known to contribute to cardiovascular and cerebrovascular diseases (Yahagi et al., 2017), renal failure (Braunwald., 2019), and other complications. Obesity is considered the leading cause of T2DM and accounts for 80–85% of the risk of developing T2DM (Minerva., 2021; Apovian et al., 2019). Several studies have reported that Wnt signaling plays a role in regulating the biological function of adipocytes (Ross et al., 2000; Bennett et al., 2002; Moldes et al., 2003; Longo et al., 2004; Wright et al., 2007). The relationship between the Wnt signaling pathways and T2DM was first documented in 2006 (Grant et al., 2006). These authors identified a genetic polymorphism of the gene encoding for transcription factor 7-like 2 (*Tcf7l2*) gene, which encodes an important transcription factor TCF4 in the Wnt signaling pathways; *Tcf7l2* is the strongest T2DM candidate gene discovered to date. The TCF7L2 protein is a key transcriptional effect or of the Wnt/β-catenin signaling pathway, which is an essential developmental pathway that negatively regulates adipogenesis (Chen et al., 2018). Subsequently, recent studies proved that dysregulation of the Wnt signaling pathways participates in the occurrence and progression of T2DM by directly influencing the differentiation and proliferation of pancreatic β-cells and the secretion and action of insulin (Nie et al., 2021). For instance, Wnt5a, a member of the Wnt family, is involved in the regulation of adipogenesis and obesity *via* the Wnt non-canonical pathway and the Wnt/β-catenin canonical pathway by inhibiting the expression of peroxidase proliferation receptor γ (PPARγ) and tumor suppressor gene C/enhancer binding protein α (CCAAT/EBPα) in adipocytes (Takada et al., 2007; Tang and Lane, 2012). In addition, Wnt5a promotes adipogenesis by binding directly to receptor tyrosine kinase-like orphan receptor 2 (ROR2) that inhibits the canonical Wnt pathway and alleviates the inhibition of adipogenesis (Mikels and Nusse, 2006). In addition, besides its potent inhibition of adipogenesis, the Wnt/β-catenin signaling pathway has been implicated in the balance between adipogenesis and myogenesis, as reported by previous studies of mesenchymal cell fate, obesity, and T2DM (Prestwich and Macdougald, 2007). The knockout of *Wnt10b in vivo* leads to an increased potential for the trans-differentiation of myoblasts into adipocytes and the acquisition of adipocyte characteristics during muscle regeneration (Taylor-Jones et al., 2002; Vertino et al., 2005). Components of the Wnt pathway are abundantly expressed in the pancreas and tissues of the small intestine, thereby playing a role in the direct or indirect regulation of the functionality of pancreatic β-cells (Cui et al., 2020). *In vitro* studies have shown that Wnt signaling molecules regulate a variety of cell cycle regulators and then promote the growth and proliferation of islet β-cells (Rulifson et al., 2007; Yoshihara et al., 2020). Disturbances in the Wnt signaling pathway may also have adverse effects on the morphology, structure, and function of pancreatic β-cells (Schinner., 2009). These observations suggest that Wnt may play an important role in the occurrence and development of diabetes.

## Wnt Signaling Pathway and DN

DN is a major complication of diabetes (Kato and Natarajan, 2019), a chronic kidney disease, and an important cause of end-stage renal failure. The incidence of DN has risen rapidly over the past few decades. The evaluation of early DN patients in clinical studies found that the main manifestations of DN are a reduction in glomerular filtration rate, proteinuria, elevated arterial blood pressure, and progressively severe sodium and water retention, eventually leading to renal failure (Wada and Makino, 2013). The main pathological features of DN include mesangial expansion, podocyte damage, basement membrane thickening, and glomerular and tubular cell damage, thus leading to glomerular sclerosis and interstitial fibrosis (Hovind et al., 2001; Parving, 2001).

Studies have shown that multiple signaling pathways are involved in the progression of DN, including the transforming growth factor β (TGFβ)/Smad, PI3K/Akt, the p38 mitogen-activated protein kinase (p38MAPK), and the Janus kinase/signal transducers and activators of transcription (JAK/STAT) pathways (Brosius et al., 2010; Zhou et al., 2012). The Wnt canonical signaling is being activated during kidney injury while it is relatively inactive in normal adult kidney (Zuo et al., 2018; Malik et al., 2020). The Wnt canonical pathway has been implicated as a dominant regulator in the development of DN (Guo et al., 2019); meanwhile, the Wnt non-canonical pathway has begun to attract attention as a potential mechanism for DN pathogenesis (von Toerneet al., 2009; Babayeva et al., 2011).

## Mechanistic Actions of the Wnt Pathway in the Promotion of Epithelial to Mesenchymal Transition in Podocyte

Podocytes are highly specialized epithelial cells that cover the outer layer of the glomerular basement membrane (GBM). Podocytes play an important role in regulating glomerular function, including the formation and renewal of the basement membrane, and maintaining the structural stability of the capillary loop. Podocytes are terminally differentiated cells that no longer proliferate. Therefore, the limited supply of primary podocytes hinders the replacement of damaged podocytes. These differentiated cells are composed of three segments with different morphologies and functions: the cell body, the main process, and the extensor foot process (FP) (Yiu., 2018). FPs are rich in filamentous actin (F-actin) and cover the surface of the GBM in the form of finger-like crosses. Podocytes use proteoglycans and the cell matrix to adhere to the GBM (Sever and Schiffer, 2018). The FPs of adjacent podocytes are connected by a continuous adhesive structure referred to as the slit diaphragm (SD). The FPs combine to form a huge filtration surface that covers the glomerular filtration barrier (GFB). The extracellular SD is connected to the highly dynamic intracellular cytoskeleton by adaptor proteins. Collectively, The GFB consists of three layers, which is the fenestrated endothelium that is covered by the glycocalyx, the podocytes and the intervening glomerular basement membrane and play an important role in controlling the influx and efflux of proteins (Garsen et al., 2014). Therefore, podocyte damage and dysfunction inevitably play an important role in the pathogenesis of DN.

Recent studies have shown that podocyte injury is an early and critical stage of the progression of DN (Zhang et al., 2015). After receiving harmful stimuli, such as high glucose (Dai et al., 2020; Li and Siragy, 2014) or genetic mutation (Bork et al., 2020), the podocyte is known to respond differently according to the severity and duration of the injury. Previous studies have shown that podocyte tend to undergo hypertrophy or autophagy in the process of DN and that this occurs *via* the mTOR signaling pathway (Bork et al., 2020). Hypertrophy is the process by which cells increase their volume to compensate for the loss of function. Autophagy is the process by which excess or abnormal cellular components are removed by lysosomal self-degradation under normal physiological conditions. Podocytes undergo de-differentiation and Epithelial-to-Mesenchymal Transition (EMT) after progressive or severe damage. As a result, podocytes acquire mesenchymal characteristics, such as the expression of mesenchymal markers; this leads to the symptoms of clinical proteinuria and the loss of epithelial characteristics. This conversion might represent the initial cause of proteinuria (May et al., 2014). Podocytes that have been subjected to severe or chronic damage gradually die or become detached from the GBM; this leads to podocytosis, which aggravates proteinuria and glomerulosclerosis (Campbell and Tumlin, 2018).

The Wnt canonical cascade and WNT protein function in the event of podocyte injury. The presence and expression of β-catenin are necessary in normal podocytes (Zhou et al., 2017). According to a previous study, the abnormal up-regulation of the Wnt/β-catenin signaling pathway leads to podocyte damage and dysfunction (Dai et al., 2009) (Supplementary Figure S2). Wilm’s tumor 1 (WT1) is a nuclear protein that is uniquely expressed in podocytes and is generally considered as a podocyte marker (Zhou et al., 2015). Zhou et al. reported that the abnormal activation of the Wnt/β-catenin pathway inhibits WT1-mediated gene expression and induces podocyte de-differentiation and mesenchymal transformation. In contrast, blocking the Wnt/β-catenin pathway restores WT1-mediated gene silencing, thus maintaining the integrity of podocytes (Zhou et al., 2015). Abnormally activated Wnt signal result in decreased expression of podocyte differentiation markers, and increased expression of parietal epithelial cell (PEC) specific markers simultaneously. Indicating that Wnt/β-Catenin signal may mediate the transformation of PEC to podocytes (Kato et al., 2011; Kato and Susztak, 2012; Ni et al., 2021). SD component connects FPs of adjacent podocytes and function in maintaining the structural integrity of SD and podocytes (Zhou et al., 2015). Renin is a key protein that is associated with SD and can be inhibited by Snail, a common transcription factor that can promote EMT by inhibiting the expression of E-cadherin (Matsui et al., 2007; Kang et al., 2010). Li et al. demonstrated that when β-catenin is activated there are reductions in the content of renin and the un-regulation of Snail, thus resulting in significant reduction in expression and reorganization of podocyte cytoskeleton protein, F-actin, in response to HG (Li and Siragy, 2014). Furthermore, TGFβ1 has also been shown to promote the activation of the Wnt/β-catenin signaling pathway and down-regulate the levels of Snail and other substances that can lead to podocyte damage (Wang et al., 2011). Therefore, aberrant activation of the Wnt/β-catenin signal reduces podocyte adhesion and migration by regulating a variety of downstream molecules that lead to EMT in podocytes.

However, it has been reported that in cultured podocytes, a reduction in Wnt/β-catenin activity led to an increase in the expression of podocyte differentiation markers and enhanced podocyte activity (Supplementary Figure S2). Podocytes are therefore more susceptible to kidney injury -induced apoptosis. The study from the same group involving transgenic diabetic mice, showed that a reduction in β-catenin expression levels resulted in an inactive Wnt pathway and aggravated the symptoms of proteinuria and podocyte injury. Over-expression of Dickkopf-1 (DKK-1) in the transgenic diabetic mice resulted in more severe injury to the podocytes when compared with the control group (Kato et al., 2011). In other words, the reduction in the activity of the Wnt pathway can also lead to abnormal podocyte function.

In addition, the Wnt/PCP pathway also plays a pivotal role during podocyte injury. The Wnt/PCP pathway either activates the RhoA/ROCK pathway or signals through JNK and is thought to regulate changes in the cytoskeleton (Endo et al., 2005; Liang et al., 2007) (Supplementary Figure S2). In a previous study, Babayev et al. (Babayeva et al., 2011) used Wnt5a, a non-canonical ligand of the Wnt/PCP pathway, to treat cultured human and mouse podocyte cell lines *in vitro*. The transcription of Vangl2, Frizzled, Van Gogh, Dvl, Fat and Daam1, were then detected in both cell lines by real-time polymerase chain reaction (RT-PCR) (Wolff and Rubin, 1998; Usui et al., 1999; Yang et al., 2002; Sato et al., 2010), thus demonstrating the activation of the Wnt/PCP pathway in podocytes. Subsequently, anti-renin antibody and phalloid in staining was used to detect the distribution of renin in a human podocyte cell line after Wnt5a treatment (Babayeva et al., 2011). These data suggested that the Wnt/PCP pathway may change the distribution of renin, thereby promoting podocyte damage (Babayeva et al., 2011). A study using single-cell sequencing from human glomerulonephritis and chronic interstitial nephritis specimens showed that the expression of the *JunB* gene in podocytes was upregulated. JunB is a component of the activating protein-1 (AP-1) transcription factor. The upregulation of JunB was possibly related to the activation of Wnt/PCP signaling in the human nephrotic samples.

Focal segmental glomerulosclerosis (FSGS) is a common forms of genetically inherited chronic kidney diseases (CKDs). (Rosenberg and Kopp, 2017). Wnt1 is markedly upregulated and Wnt/β-catenin signal is activated in glomerular cells of FSGS patients (Dai, et al., 2009). Members of the transient receptor potential canonical (TRPC) channel family, including TRPC6 channels, are shown as key contributors in the pathogenesis of renal and cardiovascular diseases (Abramowitz_ and Birnbaumer, 2009; Ma et al., 2016; Ilatovskaya and Staruschenko, 2015). TRPC belongs to the superfamily of TRP cation channels that are non-selectively permeable to calcium (Abramowitz and Birnbaumer, 2009; Ilatovskaya and Staruschenko, 2015). TRPC6 is a SD-associated protein in podocytes that is involved in regulating glomerular filter function, and gain-of-function mutation in TRPC6 has been associated with the onset of the familial FSGS (Reiser et al., 2005; Winn et al., 2005; Moller et al., 2007). Overexpression of wild-type TRPC6 appears sufficient to cause proteinuria in healthy mice. There is possibility that TRPC6 cross talk with Wnt signaling in controlling podocyte dysfunction, inflammation and fibrosis (Li et al., 2013) (Supplementary Figure S2).

It is reported that HG induced time dependent up-regulation of TRPC6 and activation of the canonical Wnt signaling pathway in mouse podocytes. Blockade of the Wnt signaling pathway by DKK-1 resulted in effective reduction of TRPC6 up-regulation and amelioration of podocyte apoptosis. This data present a mechanistic linkage between Wnt/β-catenin activation, TRPC6 induction and podocyte injury (Li et al., 2013). However, in a study with transgenic mice model, Wang et al. reported that NFAT activation may be a key intermediate step in the pathogenesis of mutant TRPC6-mediated FSGS. They observed upregulation of Wnt6 and Fzd9 in the mutant glomeruli before the onset of significant proteinuria, suggesting a potential role for Wnt signaling in the pathogenesis of NFAT-induced podocyte injury and FSGS. The up-regulation of Wnt6/calcineurin/NFAT axis suggesting the potential activation of the Wnt/Ca^2+^ pathway in mutant TRPC6-mediated podocyte injury (Wang et al., 2010; Nijenhuis et al., 2011; Ma et al., 2019). Thus, both the canonical and non-canonical Wnt/Ca^2+^ pathways may play a role during the process of podocyte injury.

In the Wnt canonical pathway, podocytes can be damaged whether the level of β-catenin is increased or decreased. The Wnt may play a dual role in the regulation of podocytes in the process of DN. Abnormal activation of Wnt/PCP pathway and Wnt/Ca^2+^ can also lead to podocyte injury. The specific mechanisms associated with the Wnt signaling in the promotion of the epithelial to mesenchymal transition in podocytesis shown in Supplementary Figure S2.

## The Role of Wnt Signal Mediation in Mesangial Cell Injury

Mesangial cells (MCs) are contractile cells that make up the central stalk of the glomerulus. On the capillary lumen side, the mesangial cells make direct contact with the glomerular endothelium without the intervention of the basement membrane; however, cell protrusions can extend to the space between the endothelial cells and the basement membrane (Sakai and Kriz, 1987) or the capillary lumen through the gap between endothelial cell (Zhao, 2019), the mesangial cells form a supporting framework that maintains the structural integrity of the glomerular capillary network (Kurihara and Sakai, 2017). In addition, their contractile properties enable mesangial cells to change capillary flow in the glomerulus and the glomerular ultrafiltration surface area, thereby coordinating the single-nephron glomerular filtration rate to play a key role in the glomerular filtration barrier (Rabelink et al., 2015). In addition, mesangial cells can also communicate with endothelial cells by releasing cell growth factors such as platelet-derived growth factor (PDGF), renin, TGF, and other biologically active substances that are crucial for the stability of glomerular endothelial function.

MCs are susceptible to stimulation by high levels of glucose. Evidence shows that in patients with diabetic nephropathy, the damage incurred by MCs in response to hyperglycemia is multidirectional. HG can induce apoptosis in MCs (Supplementary Figure S3). HG stimulates the formation of Ras/Rac1-dependent superoxide; this then subsequently inhibits Wnt proteins, including WNT4, WNT5a, and WNT6 (Tung et al., 2018). In a previous study, diabetic rats were introduced to superoxide dismutase-conjugated propylene ethyl glycol (SOD-PEG) to eliminate superoxide; analysis showed that the harmful effects of free radicals on MCs had been improved. HG induces the activation of GSK-3β leading to β-catenin instability and degradation; this then induces MC apoptosis by promoting the cleavage of caspase-3 and polyADP-ribose polymerase (PARP) (Xiao et al., 2013). A previous study proved that the transfection of WNT4, WNT5a, or stable β-catenin (S33Y) in MCs can inhibit the activation of GSK-3β and increase the stability of nuclear β-catenin, thereby reducing the level of apoptosis in MCs (Lin et al., 2006). Thus, the Wnt signaling is involved in the process of MC damage. The clinical use of medicines such as simvastatin (SIM) (Lin et al., 2008) and spironolactone (SPI) (Zhu et al., 2013) enhance the secretion of Wnt5a protein in MCs when administered to patients with DN. The increased secretion of Wnt5a protein promoted the translocation of β-catenin to the nucleus, thereby increasing the HG-induced inhibition of Wnt/β-catenin to protect glomerular MC injury, thereby generating a beneficial effect for patients with diabetic nephropathy. DKK-1 mediates the HG-induced reduction of β-catenin in the nucleus while the knockout of DKK-1 inhibits the expression of TGFβ and fibronectin (FN) in renal MCs, thereby reducing glomerular volume and the deposition of mesangial matrix (Lin et al., 2010). scRNA sequencing results of specimens from patients undergoing kidney transplantation, or patients with glomerulonephritis, malignant renal tumors, or chronic interstitial nephritis, revealed that the gene expression of chloride intracellular channel 1 (*Clic1*) was up-regulated (Chen et al., 2021). CLIC1 is a sensor of oxidative stress in endothelial cells (ECs). However, the mechanism by which CLIC1 mediates the regulation of endothelial dysfunction has yet to be fully elucidated. An *in vitro* study of mouse mesenchymal C3H10T1/2 cells found that CLIC1 responds to Wnt stimuli (Yang et al., 2009). Therefore, the high expression of CLIC1 in nephrotic specimens may potentially be regulated by Wnt signaling.

However, on the other hand, it has been reported that high levels of glucose can induce MC proliferation, inflammation, and deposition of collagen, laminin, and FN through the Wnt/PCP pathway. HG promotes the generation of reactive oxygen species and thus activates nuclear factor kappa-light-chain-enhancer of activated B cells (NF-κB) signal accompanied by overproduction of intercellular TGFβ1 and FN (Supplementary Figure S3). Berberine ameliorates experimental diabetes-induced renal inflammation and FN by inhibiting the activation of RhoA/ROCK signaling (Xie et al., 2013). In MCs, G protein-coupled bile acid receptor Gpbar1 (TGR5) activation or overexpression significantly suppressed FN and TGFβ1 protein expression, as well as NF-κB and RhoA/ROCK activation induced by HG or the transfection of constitutively active RhoA (Xiong et al., 2016). In another study, in cultured MCs, exposure to HG activated NF-κB nuclear translocation and DNA binding activity was attenuated by the ROCK inhibitor Y27632 or a dominant-negative RhoA mutant, thus indicating that RhoA/ROCK signaling regulates the high glucose-activated NF-κB pathway. These data further confirmed that high glucose affects MC injury through the Wnt/PCP pathway (Xie et al., 2013).

Wnt canonical signaling, together with the non-canonical PCP cascade, mediates MC damage in multiple directions when exposed to hyperglycemia. The role of Wnt signal mediation in MC injury under high glucose stimulation is shown in Supplementary Figure S3.

## Wnt Signaling Pathway and Renal Fibrosis

Renal interstitial fibrosis is a common pathway for renal diseases to develop to end-stage renal failure and is one of the most recognized pathological features of DN (Kavanagh et al., 2011). Renal fibrosis refers to the fibrosis of cells inside the kidney, thus leading to renal damage and death. The primary mechanism underlying this effect is the deposition of extracellular matrix (ECM); this gradually results in renal sclerosis, scarring, the eventual loss of renal function (Zeng et al., 2019), as well as epithelial-mesenchymal cell trans-differentiation. The occurrence of renal fibrosis is related to a variety of cytokine signaling pathways. The Wnt signaling pathway and other signaling pathways are known to participate jointly in renal fibrosis.

WNT ligand proteins play a key role in renal genesis and renal disease. During renal development, WNT4 promotes the regeneration of renal tubular epithelial cells by regulating cyclinD1 and cyclinA (Terada et al., 2003; Kawakami et al., 2013) and by stimulating the differentiation of unshaped mesenchymal cells into epithelial cells. It has been reported that *Wnt4* and *Wnt11* genes are expressed in the posterior kidney and encode secretory glycoproteins that are important mediators of renal EMT. Nguyen et al. found that the normal expression pattern of Wnt4 and Wnt11 was disrupted in a model of posterior renal obstruction, thus interfering with the normal transformation process in the developing kidney by maintaining mesenchymal components and inducing EMT (PNguyen, et al., 1999). Surendran et al. (Surendran et al., 2002) detected an increased number of MCs and enhanced WNT4 protein levels in a rat model of unilateral ureteral obstruction; these increases in expression were proportional to the high content of collagen I and a-smooth muscle actin, thus indicating that WNT4 can induce the formation of fibroblasts *in vitro* (Liu et al., 2020a). In conclusion, a variety of WNT ligand proteins participates in renal fibrosis and act by regulating protein expression.

The downstream target genes of the Wnt canonical pathway are all involved in renal fibrosis, including fibronectin 1 (FN1), Cluster of differentiation 44 (Cd44), fibroblast specific protein-1 (FSP-1), and matrix metalloproteinase-7 (MMP-7) (Pishvaian and Byers, 2007). Studies involving a mouse model of renal transplantation showed that Cd44 expression was increased in the renal interstitium, and that the FN1 protein content was also increased in the renal interstitium; the expression levels of these genes were closely correlated with renal tubule interstitial fibrosis. The FSP-1gene aggravates the occurrence and development of renal fibrosis by activating myofibroblasts (Luo et al., 2018). MMP-7 is a secreted zinc- and calcium-dependent endopeptidase that degrades a variety of extracellular matrix substrates, including E-cadherin and renin (Liu et al., 2020b). Uterine sensitization-associated gene-1 (USAG-1) is one upstream regulator of the Wnt canonical signaling. It is highly expressed as a cancer suppressor in the kidney (Blish et al., 2009). Recent study show that USAG-1 protecting renal tubules from interstitial fibrosis caused by ischemia-reperfusion injury or drug toxicity injury by regulating the Wnt signal and other related pathways (Tanaka et al., 2008; Lu et al., 2019; Badshah et al., 2019; Blish,et al., 2009; Li et al., 2021).

Studies have demontrated that the Wnt/Ca^2+^ pathway also participate in the formation of renal fibrosis. It is well known that the Wnt/Ca^2+^ pathway plays a key role during cardiomyocyte hypertrophy and pathological myocardial remodeling in the heart (Bourajjaj et al., 2008). However, reports relating to the Wnt/Ca^2+^ pathway in renal injury are rare. Von Toerne et al. used a rat model of kidney transplantation to investigate the main pathophysiological processes underlying renal failure. With the development of chronic injury, the expression levels of TGFβ and canonical Wnt signaling target molecules (FN1, Cd44, MMP7, and Nos2) were all upregulated and associated with the progression of kidney injury. Importantly, the central components of the Wnt/Ca^2+^ signaling pathway are also significantly altered with the progression of chronic injury. The involvement of the Wnt/Ca^2+^ pathway in renal fibrosis was confirmed by increased expression levels of Wnt6, Wnt7a, protein kinase C(PKC), CaMKII, NFAT transcription factors, and the target gene vimentin. Both collagen I and collagen III synthesis are enhanced followed by increased phosphorylation of CaMKII, which is correlated with progressive damage and fibrosis in transplanted kidneys. In contrast, alteration of the Wnt/PCP pathway was not observed in this model (von Toerne et al., 2009).

The Wnt pathway acts in synergy with other pathways such as the receptor tyrosine kinase/Ras/mitogen-activated protein kinases (RTK/Ras/MAPK) pathway and the phosphatidylinositol3-kinase/integrin-linked kinase/protein kinase B (PI3K/ILK/PKB) pathway. These pathways jointly regulate the expression of snail and E-adhesion protein to promote renal fibrosis (Rosivatz et al., 2002; Behrens, 2005). The Wnt canonical pathway also mediates the formation of renal fibrosis in association with TGFβ signaling. TGFβ induces the expression of MMP-7 which indirectly promotes renal fibrosis through EMT by damaging the epithelial integrity of the renal tubule. Induced matrix metalloproteinase-7 (MMP-7) can then activate MMP-2 and MMP-9 by proteolysis, thus leading to renal fibrosis by degrading collagen IV and promoting EMT (Cheng and Lovett, 2003; Cheng et al., 2017).

Wnt ligands, especially Wnt1-inducible signaling pathway protein-1 (WISP-1), are involved in renal fibrosis associated with the TGFβ pathway. Elevated levels of WISP-1 in the renal tubular epithelium of rats were shown to increase the production of fibrotic substances induced by TGFβ (Yang et al., 2020). By upregulating the nuclear expression of bone marrow mesenchymal genes (such as Zeb1, Pai1, and α-SMA) (DiGregorio, et al., 2020), WISP-1 can also promote renal fibrosis and mediate TGF signaling. TGFβ activates Wnt signals by down-regulating the production of DKK-1 (Akhmetshina et al., 2012). Besides the TGF pathway, Wnt can also interact with the PPAR pathway. Peroxisome proliferator-activated receptor-α (PPARα) is known to negatively regulate the Wnt pathway. Cheng et al. reported that HG upregulated the expression of PPARα in human renal proximal tubule cells (HRPTCs). The overexpression of PPARα was also shown to attenuate the expression of connective tissue growth factor (CTGF) and FN induced by HG, thus demonstrating the anti-fibrotic activity of PPARα in diabetic renal cells (Cheng et al., 2016).

Renal fibrosis is one of the special symptoms of DN. The abnormally regulated Wnt signaling that causes damage to podocytes or MCs will eventually lead to renal fibrosis. In addition, WNT4 and WNT11 cannot only directly induce renal mesenchymal differentiation; they can also induce renal fibrosis by regulating downstream target genes related to fibrosis *via* the Wnt signaling pathway. Therefore, studying the expression of Wnt protein is also of great importance to DN. In addition, the non-canonical Wnt/Ca^2+^ pathway is also involved in the process of renal fibrosis and acts with other pathways, such as the RTK/Ras/MAPK pathway. The TGFβ and PPARα pathways interact with each other and regulate renal fibrosis in a coordinated manner.

## Conclusion and Remarks

Diabetic nephropathy (DN) caused by diabetes mellitus is one of the major causes of end-stage renal failure. The pathogenesis of DN characterized by mesangial expansion, podocyte injury, basement membrane thickening, glomerular and renal tubular cell injury is complex and diverse, all of those damage to renal tissues eventually lead to glomerulosclerosis and interstitial fibrosis. It has been proved that Wnt signal plays an important role in the occurrence and development of DN. Wnt canonical signal plays a key role in the occurrence and development of DN. It has been demonstrated that exogenous Wnt1 prevents acute kidney injury and subsequent chronic renal injury (Hong et al., 2021). Supplement of the reduced Wnt signal and neutralizing abnormal Wnt cascade activation may be potential strategies for therapeutic intervention of progressive renal diseases (von Toerne, et al., 2009; Luo et al., 2007; He et al., 2009; Li, et al., 2021). However, the involvement of Wnt Non-canonical pathway with DN progression had not been paid enough attention in details. In this review, we show that in addition to Wnt/β-catenin pathway, alteration of the non-canonical Wnt/PCP and Wnt/Ca^2+^ pathways appear to contribute to the pathogenesis of renal tissues including epithelial to mesenchymal transition in podocyte, mesangial cell injury and renal fibrosis. Given that there are multiple branches in Wnt signaling pathways, it is unclear when these branches function independently and when they are interact with each other in regulation of kidney injury. With more evidence of the non-canonical Wnt/PCP and Wnt/Ca^2+^ regulation of DN being accumulated, we believe that this information will provide a more comprehensive strategy for the clinical treatment of DN. The key mechanisms of the Wnt signaling for the regulation of DN are summarized in the flowchart 1.
