# Supplementary figures and images for: The Wnt Signaling Pathway in Diabetic Nephropathy
Source: Front Cell Dev Biol. 2022 Jan 4;9:701547. doi: 10.3389/fcell.2021.701547 (PMC8763969; doi:10.3389/fcell.2021.701547)

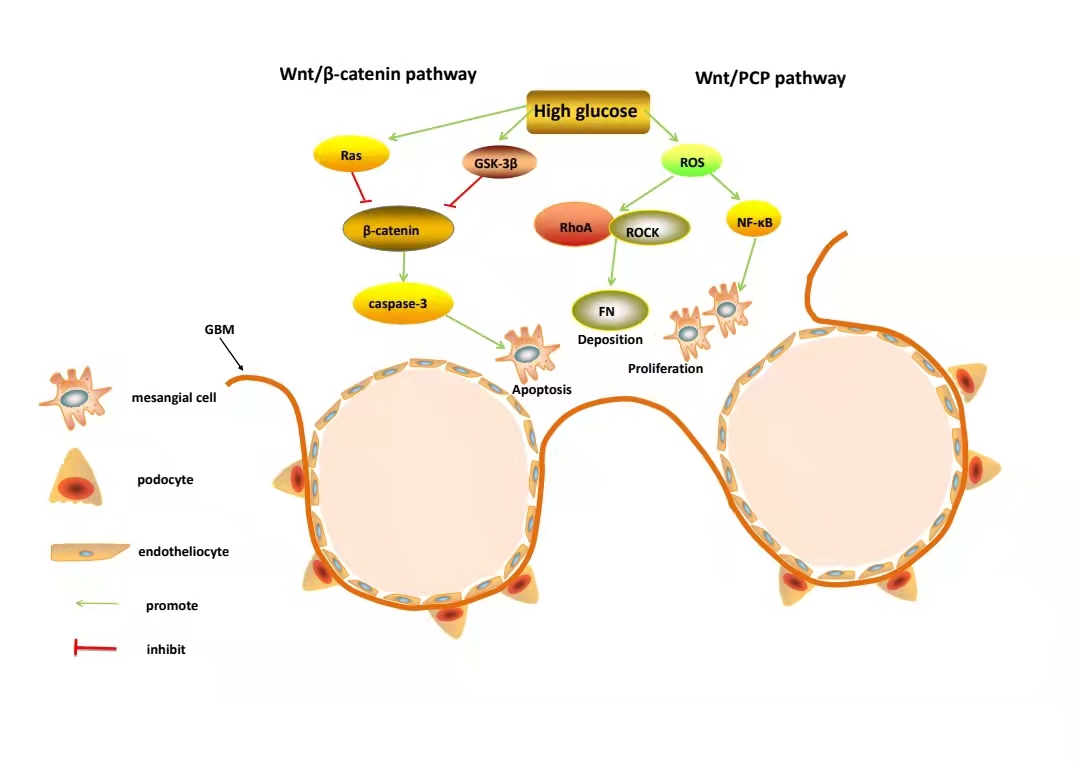

Supplement: Supplementary file 1 [file Image3.jpeg]

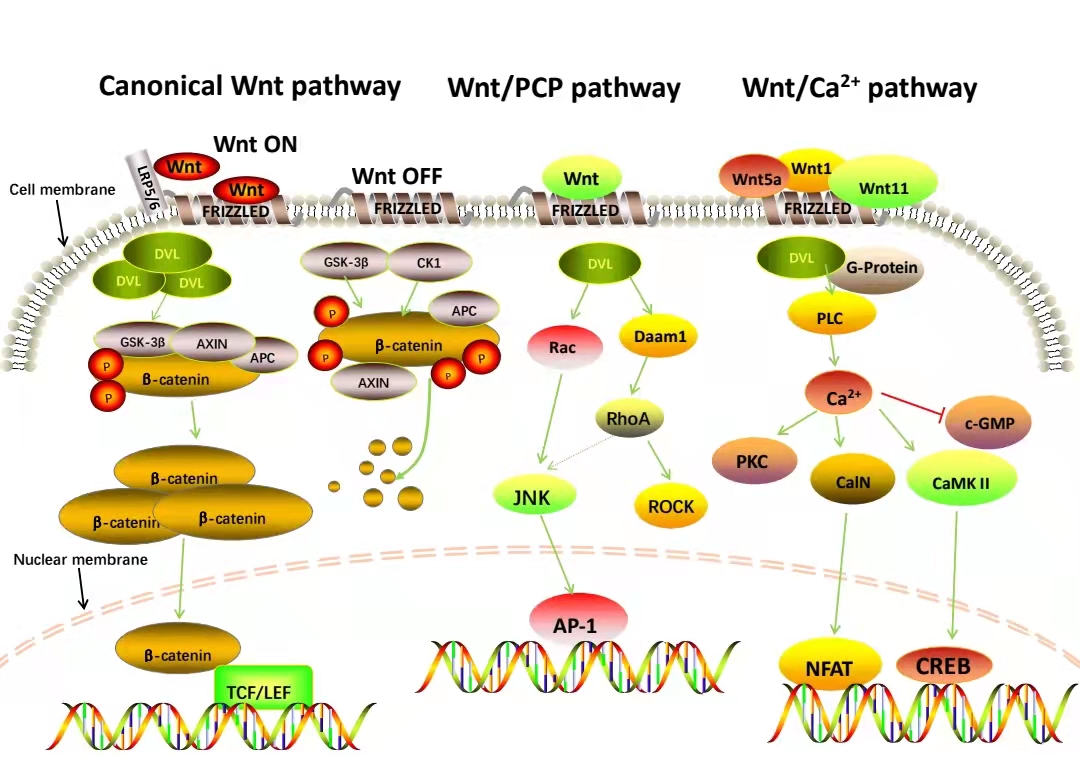

Supplement: Supplementary file 2 [file Image1.jpeg]

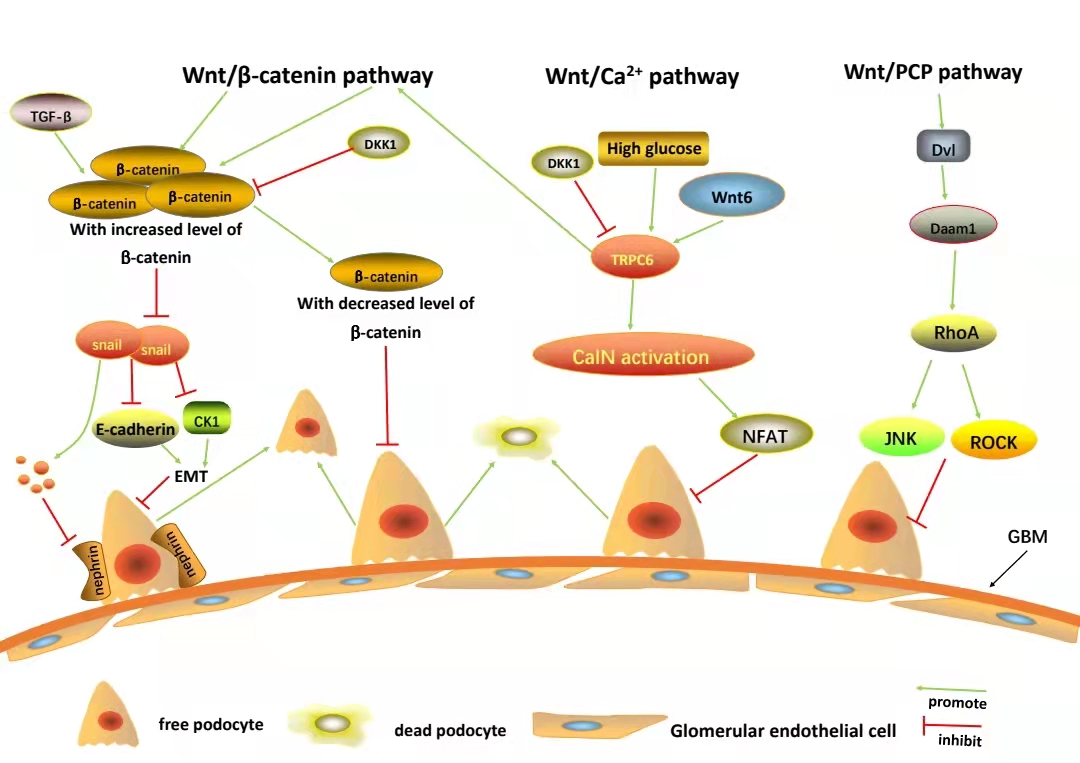

Supplement: Supplementary file 3 [file Image2.jpeg]
