# Supplementary material for: The Wnt Signaling Pathway in Diabetic Nephropathy
Source: Front Cell Dev Biol. 2022 Jan 4;9:701547. doi: 10.3389/fcell.2021.701547 (PMC8763969; doi:10.3389/fcell.2021.701547)
Supplement: Supplementary file 4 [file DataSheet2.docx]

**Figure legends**

**Figure 1. An overview of the Wnt signaling cascades**

In canonical Wnt signaling, the absence of Wnt ligands leads to the inactivation of the Wnt pathway (OFF). In this state, cytoplasmic β-catenin is trapped by a destruction complex composed of APC, Axin, CK1 and GSK-3β, which is followed by phosphorylation and degradation. The canonical pathway is activated with the binding of Wnt ligands to Fzd receptors and LRP co-receptors (ON). The formation of the Wnt-Fzd-LRP complex, together with the recruitment of Dvl, results in phosphorylation of LRP6 and the activation and recruitment of the Axin complex to the receptors, which inactivates the destruction complex. This inactivation leads to the stabilization and accumulation of β-catenin, which translocates into the nucleus. There, β-catenin forms an active complex with TCF/LEF and other transcription factors, leading to a switch on multiple cellular gene transcription events.

Non-canonical Wnt signaling is defined by β-catenin-independent signal transduction. During Wnt/PCP signaling, Wnt ligands bind to the Fzd receptor to recruit and activate Dvl. Then Dvl is recruited and Dvl-Daam-1 complex is activated, followed by JNK and ROCK activation and cytoskeletal rearrangement; Activated Dvl also enables Rac activation, which activates JNK. In the Wnt/Ca^2+^ signaling pathway, the binding of the Wnt ligands to Fzd activates the G-protein, which then activates PLC. PLC leads to an increase in intracellular Ca^2+^and the activation of CaMK II, CalN and PKC followed by CREB or NFAT transcription factor transcriptional responses respectively. APC, adenomatous polyposis coli; AP-1, activating protein-1; CaMK II, calmodulin-dependent protein kinase II; CalN, calcineurin; CK1, casein kinase 1; Daam1, dishevelled-associated activator of morphogenesis 1; Dvl, Dishevelled; GSK-3β, glycogen synthase kinase 3β; JNK, Jun N-terminal kinases; LRP5/6, low-density lipoprotein receptor 5/6; NFAT, nuclear factor of activated T cells ; PLC, phospholipase C; PKC, protein kinase C; RhoA, Ras homologue gene-family member A; ROCK, Rho-associated kinase; TCF/LEF, T cell factor/Lymphoid Enhancer Factor.

**Figure 2. The Wnt signaling in promotion of epithelial to mesenchymal transition in podocytes**

The canonical Wnt/β-catenin pathway has dual roles in podocyte injury. Podocytes received abnormal stimulation when the Wnt/β-catenin pathway is aberrantly activated or inhibited. Also, both non-canonical signaling pathways, Wnt/Ca^2+^ and Wnt/PCP participate in podocyte injury. In the Wnt/Ca^2+^ signaling pathway, the binding of the ligand to the Fzd leads to the activation of homotrimeric G protein and activates PLC which results in calcium release. Increased Ca^2+^ level activates down-stream kinases and transcription factors such as NFAT which are positive regulators of non-canonical target gene expression and negative regulators of expression of β-catenin controlled genes. In the Wnt/PCP pathway, Damm1-RhoA activates the JNK signal, which leads to changes in the distribution of renin, which subsequently affects podocyte function. CalN, calcineurin; CK1, casein kinase; Daam1, dishevelled-associated activator of morphogenesis 1; DKK1, Dickkopf-1; Dvl, Dishevelled; EMT, epithelial-mesenchymal transition; JNK, Jun N-terminal kinases; RhoA, Ras homologue gene-family member A; ROCK, Rho-associated kinase; TRPC6, Transient receptor potential channel 6; TGFβ, transforming growth factor β.

**Figure 3. The Wnt pathway and mesangial cell damage under high glucose conditions**

High glucose induces mesangial cell damage by activating both Wnt canonical and Wnt non-canonical PCP signal pathways. On the one hand, high glucose activates Ras and GSK-3β, thereby degrading β-catenin. Then caspase-3 is activated, which promotes mesangial cell apoptosis. On the other hand, high glucose induces ROS production, which stimulates RhoA, ROCK, and NF-κB that leads to events such as the proliferation of mesangial cells and the deposition of mesangial extracellular matrix. ECM, extracellular matrix; FN, fibronectin; GSK-3β, glycogen synthase kinase 3β; NF-κB, nuclear factor kappa-light-chian-enhancer of activated B cells; RhoA, Ras homologue gene-family member A; ROCK, Rho-associated kinase; ROS, reactive oxygen species.
