# Supplementary material for: The Wnt Signaling Pathway in Diabetic Nephropathy
Source: Front Cell Dev Biol. 2022 Jan 4;9:701547. doi: 10.3389/fcell.2021.701547 (PMC8763969; doi:10.3389/fcell.2021.701547)
Supplement: Supplementary file 5 [file DataSheet1.docx]

**The key mechanisms of the Wnt signaling for regulation of DN**
